# The impact of regular physical activity on fatigue, depression and quality of life in persons with multiple sclerosis

**DOI:** 10.1186/1477-7525-7-68

**Published:** 2009-07-20

**Authors:** Nicole M Stroud, Clare L Minahan

**Affiliations:** 1 School of Physiotherapy and Exercise Science, Gold Coast Campus, Griffith University, Queensland, Australia

## Abstract

**Background:**

The purpose of this study was to compare fatigue, depression and quality of life scores in persons with multiple sclerosis who do (Exercisers) and do not (Non-exercisers) regularly participate in physical activity.

**Methods:**

A cross-sectional questionnaire study of 121 patients with MS (age 25–65 yr) living in Queensland, Australia was conducted. Physical activity level, depression, fatigue and quality of life were assessed using the International Physical Activity Questionnaire, Health Status Questionnaire Short Form 36, Becks Depression Inventory and Modified Fatigue Impact Scale.

**Results:**

52 participants performed at least two 30-min exercise sessions·wk^-1 ^(Exercisers) and 69 did not participate in regular physical activity (Non-exercisers). Exercisers reported favourable fatigue, depression and quality of life scores when compared to Non-exercisers. Significant weak correlations were found between both leisure-time and overall reported physical activity levels and some subscales of the quality of life and fatigue questionnaires. Additionally, some quality of life subscale scores indicated that regular physical activity had a greater benefit in subjects with moderate MS.

**Conclusion:**

Favourable fatigue, depression and quality of life scores were reported by persons with MS who regularly participated in physical activity, when compared to persons with MS who were classified as Non-exercisers.

## Background

Multiple Sclerosis (MS) is a relapsing or progressive neurological disease with an unknown etiology and only partially effective treatment. MS can have a negative impact on both physical and psychological well being [1,2], and individuals with this disease often report lower quality of life (QoL) scores than when compared to healthy individuals [1]. Fatigue and depression levels are higher in MS patients than healthy individuals, and these conditions may negatively impact upon QoL. However, participation in regular physical activity has been suggested to positively influence feelings of fatigue [3,4] and depression [5], as well as modify QoL [6,7] in persons with MS.

Fatigue is the most common symptom reported by persons with MS [1,8], and has been negatively associated with QoL scores [9]. The Multiple Sclerosis Council for Clinical Practice Guidelines defines fatigue as *"A subjective lack of physical and/or mental energy that is perceived by the individual or caregiver to interfere with usual and desired activities" *[10]. The pathophysiological basis of fatigue in MS is complex and its precise mechanisms unresolved. Fatigue in MS may result from primary factors, related to the disease process itself, or may be secondary due to factors such as sleep disturbance and depression [10]. Fatigue may be acute or chronic in nature. Chronic fatigue is persistent, defined as being present for any amount of time, on 50 percent of days for at least 6 weeks[10]. Acute fatigue is defined as new or a significant increase in fatigue in the previous 6 weeks[10]. Some persons with MS may also experience fatigability, where they may become exhausted after completing a period of physical activity [8]. Patients may also experience motor fatigue following a period of physical activity which may lead to symptom exacerbations [11], for example although not present at rest a subject may develop footdrop following a period of physical activity. This symptom exacerbation is temporary and will subside with a rest period [12]. Considering the symptoms, it is reasonable to suggest that fatigue, fatigability and symptom exacerbations deter individuals with MS from participating in physical activity. Nevertheless, studies have reported decreased fatigue levels in persons with MS following participation in regular physical activity [3,13]. A decrease in the level of chronic fatigue and the ability to tolerate higher levels of activity (reduced fatigability) following a program of regular exercise might lead to improvements in QoL in persons with MS.

Depression is commonly observed in persons with MS, [14,15] and has been negatively associated with QoL scores [16,17]. Participation in regular physical activity is a potential moderator of depression. Cross-sectional analyses in non-MS populations suggest that individuals who participate in regular exercise are less likely to suffer from depression [18]. If regular physical activity also positively influences depression in persons with MS, then it follows that associated improvements in QoL may be observed.

The present study compared fatigue, depression and QoL scores in persons with MS who did and did not regularly participate in physical activity. It was hypothesised that persons with MS who participated in regular physical activity would report favourable fatigue, depression and QoL scores when compared to those with MS who were classified as Non-exercisers.

## Materials and methods

### Participants

A postal survey of adult men and women aged 18–65 yr diagnosed with MS was conducted over a 6-month period. Participants were volunteers who responded to a "call for participants" mail-out sent to patients from two local databases. Three hundred randomly selected patients from a total of approximately 1000 individuals living in South-East Queensland, Australia listed on the Multiple Sclerosis Society of Queensland's patient database, and 118 MS patients listed on a database at the Gold Coast Hospital, Queensland, Australia were asked to participate. Of the 418 individuals invited to participate, 130 individuals (31%) returned the questionnaires. Seven returned questionnaires were not included due to incomplete responses and two subjects were excluded as activity levels reported were not representative of the subjects' usual activity levels. Therefore, the responses of 23 men and 98 women with MS were included in the results of this study.

### Procedures

The Human Ethics Research Committee, Griffith University, Queensland, Australia granted ethical approval for this study. A background information questionnaire provided information on demographic and disease characteristics including: sex, age, year of MS diagnosis and disease course. The Disease Steps Scale (DSS) and Multiple Sclerosis Impact Scale (MSIS-29) assessed disease severity. The DSS is an ordinal scale that asked the subjects to indicate what characteristics best represented their situation. A score of 0 = normal; 1 = mild disability, mild symptoms or signs; 2 = moderate disability, visible abnormality of gait; 3 = early cane, intermittent use of a cane; 4 = late cane, cane dependant; 5 = bilateral support; 6 = confined to a wheelchair. Although the DSS is a self administered questionnaire, it has been found to correlate significantly (r = 0.944) with the Expanded Disability Status Score (a neurologist assessed measure of disease severity) and is recommended as an alternate measure of disability status [19].

The MSIS-29 is a twenty-nine itemed questionnaire that assesses the individual's view of how their MS has impacted upon their daily functioning during the previous 2 weeks. Responses for each of the 29 items in the questionnaire were scored as follows: 1 = not at all, 2 = a little, 3 = moderately, 4 = quite a bit, 5 = extremely. The MSIS-29 is a reliable and valid measure of disease impact, and is suggested to be a useful and responsive outcome measure in clinical research [20-22].

The International Physical Activity Questionnaire (IPAQ) was used to classify subjects as either '*Exercisers' *or *'Non-exercisers'*. The IPAQ quantifies physical activity performed in the preceding 7 days. A total physical activity score is derived based on total activity time and intensity,. The total physical activity score, reported in metabolic equivalents (MET)-min·wk^-1^, is a combination of scores reported in four domains: transportation, work, domestic-garden, and leisure. Subjects were classified as '*Exercisers' *if they completed at least two, 30-min exercise sessions·wk^-1 ^during their leisure time or had a physical activity score, in the leisure domain of the IPAQ, greater than 600 MET-min·wk^-1 ^[23].

The Health Status Questionnaire Short Form-36 (SF36) assessed QoL. The SF36 is a widely used QoL measure that provides scores for eight dimensions: physical functioning (limitations in physical functioning due to health problems), role-physical (limitations in usual activities because of physical health problems), bodily pain, general health, vitality (energy and fatigue), social functioning (limitations in social functioning due to physical or emotional problems), role-emotional (limitation in usual activities due to emotional problems) and mental health (psychological distress and well-being) [24]. Combining the SF36 scales produces two summary scales: i) a physical component summary score, and ii) a mental component summary score; on all scales higher scores indicate a higher QoL. The SF36 has been found to have good reliability and validity in the general population [25], patients undergoing renal replacement therapy [26] and patients with cervical spondylotic myelopathy [27].

The Beck's Depression Inventory (BDI) assessed depression. The BDI is a twenty-one item questionnaire asking patients how they have felt over the past 7 days. Each question is scored between 0–3, with higher scores indicating more severe depression [28]. The BDI has been found to be a valid measure of depression in persons with MS [29].

Fatigue was assessed using the Modified Fatigue Impact Scale (MFIS) and provides an indication of fatigue experienced by an individual in three domains; physical, cognitive, and psychosocial. The independent scores can be analysed separately or as a combined score to give a general assessment of fatigue. Higher scores indicate that fatigue has a greater impact on the individual. The MFIS has been suggested as a useful measure of fatigue in MS research and clinical practice [30].

### Data analysis

Data was analysed using the statistical software package SPSS version 14.0. Independent t-tests between subjects classified as Exercisers and Non-exercisers were performed for age, years since MS diagnosis, MSIS-29, BDI and each subscale of the SF36 and MFIS. Chi-square analyses were performed between Exercisers and Non-exercisers for sex, disease course and disease severity. Pearson's bivariate correlations between both the overall and leisure IPAQ score and the BDI, and all subscales of MFIS and SF36 were performed. Univariate analysis between disease severity, exercise status and the BDI and all subscales of the MFIS and SF36 were conducted. Statistical significance was accepted at p ≤ 0.05.

## Results

### Demographic and clinical characteristics

In this sample population, 52 of 121 (43%) subjects were classified as Exercisers. Table 1 presents selected demographic and clinical characteristics. No significant difference in age, sex, years since MS diagnosis or disease severity was observed between Exercisers and Non-exercisers. Exercisers reported significantly lower scores on the MSIS-29 (t = -3.99, p < .001) and disease course was significantly different (χ^2 ^(4, N = 121) = 13.80, p = 0.008) between Exercisers and Non-exercisers.

**Table 1:** Subject demographic and clinical characteristics

	Exercisers n = 52	Non-exercisers n = 69	Total MS sample n = 121
Age (yr)	50 ± 10	50 ± 11	50 ± 10
Sex (% male)	23.1	15.9	19.0
Disease duration (yr)	12 ± 8	11 ± 8	12 ± 8
Disease Steps Score (%)			
0	15.4	8.7	11.6
1	26.9	24.6	25.6
2	17.3	13.0	14.9
3	5.8	11.6	9.1
4	19.2	17.4	18.2
5	11.5	11.6	11.6
6	3.8	13.0	9.1
MSIS-29	61 ± 18**	77 ± 26	70 ± 24
Disease Course (%)*			
Relapsing-remitting	51.9	55.1	53.7
Secondary progressive	23.1	13.0	17.4
Primary progressive	1.9	17.4	10.7
Progressive relapsing	0.0	4.3	2.5
Unknown	23.1	10.1	15.7

Results are presented as mean ± standard deviation unless otherwise indicated. MSIS-29: Multiple Sclerosis Impact Scale-29. ** Exercisers significantly different to Non-exercisers, p < 0.001. * Exercises significantly different to Non-exercisers, p < 0.05.

### Fatigue, depression and quality of life scores

**Figure 1:**
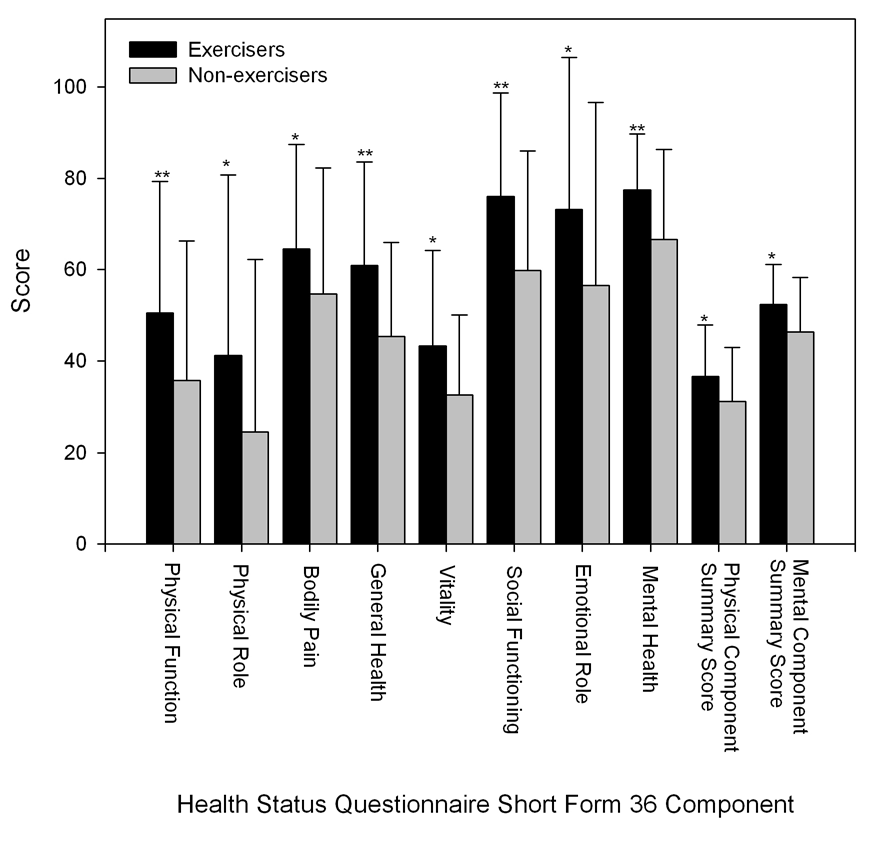
**Quality of life scores in people with multiple sclerosis**. Error bars represent standard deviations. Higher scores represent more favourable perceived quality of life. Exercisers represent individuals who reported participating in at least two, 30-min exercise sessions·wk^-1^, or had a physical activity score in the leisure domain of the International Physical Activity Questionnaire greater than 600 MET-min·wk^-1^. * Exercisers significantly different from non-exercisers, p < 0.05. ** Exercisers significantly different from Non-exercisers, p < 0.001.

**Figure 2:**
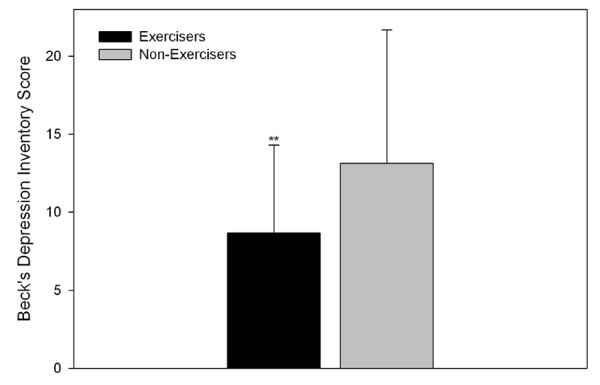
**Depression scores in people with multiple sclerosis**. Error bars represent standard deviations. Higher scores represent greater depression levels. Exercisers represent individuals who reported participating in at least two, 30-min exercise sessions·wk^-1^, or had a physical activity score in the leisure domain of the International Physical Activity Questionnaire greater than 600 MET-min·wk^-1^. ** Exercisers significantly different from Non-exercisers, p < 0.001.

**Figure 3:**
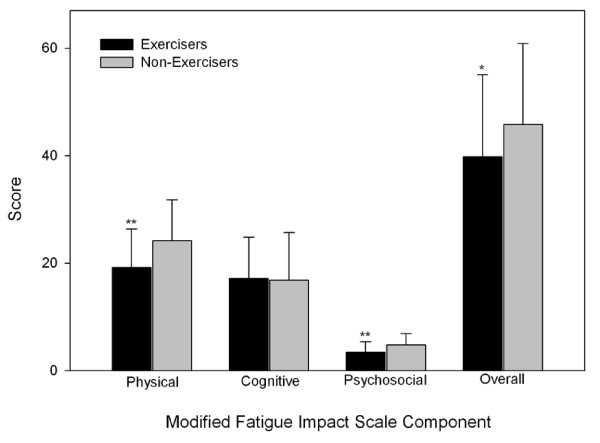
**Fatigue scores in people with multiple sclerosis**. Error bars represent standard deviations. Higher scores represent greater fatigue levels. Exercisers represent individuals who reported participating in at least two, 30-min exercise sessions·wk^-1^, or had a physical activity score in the leisure domain of the International Physical Activity Questionnaire greater than 600 MET-min·wk^-1^. * Exercisers significantly different from non-exercisers, p < 0.05. ** Exercisers significantly different from Non-exercisers, p < 0.001.

Exercisers had significantly higher scores on all scales of the SF36 when compared to Non-exercisers (Figure 1). The BDI (Figure 2), as well as the Physical and Psychosocial components, and overall score of the MFIS (Figure 3) were significantly lower in the Exercisers. There was no significant difference between Exercisers and Non-exercisers on the cognitive component of the MFIS.

### The impact of disease severity on fatigue, depression and quality of life

**Figure 4:**
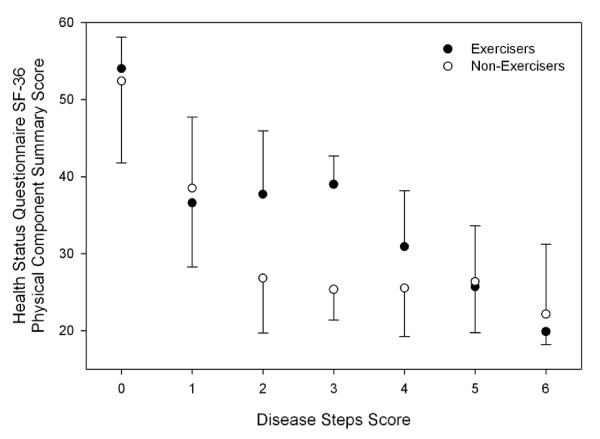
**Health Status Questionnaire Short Form-36 Physical Component Summary Score across disease severity in people with multiple sclerosis**. Error bars represent standard deviations. Higher scores represent more favourable perceived quality of life. Exercisers represent individuals who reported participating in at least two, 30-min exercise sessions·wk^-1^, or had a physical activity score in the leisure domain of the International Physical Activity Questionnaire greater than 600 MET-min·wk^-1^.

**Figure 5:**
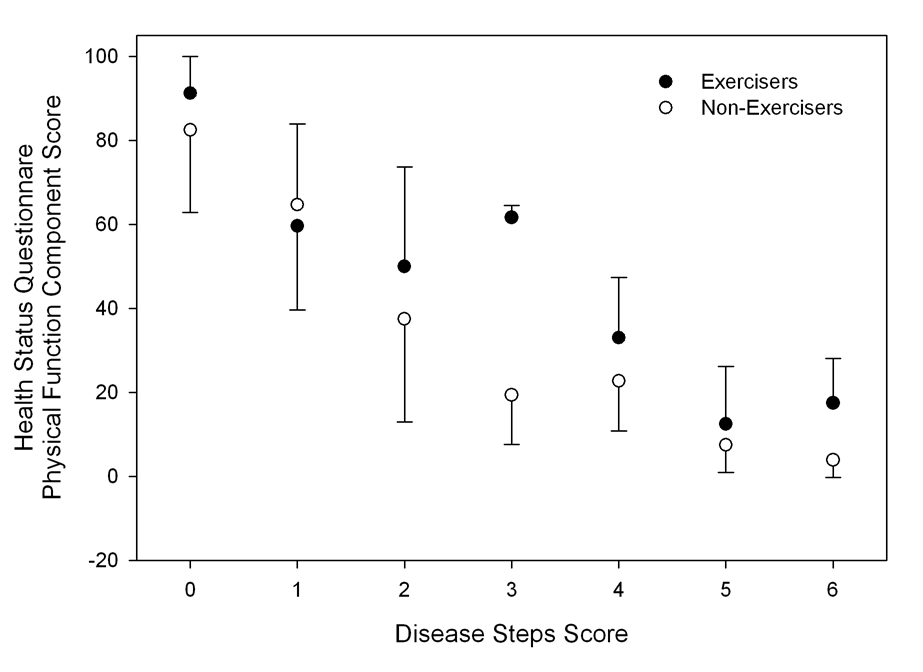
**Health Status Questionnaire Short Form-36 Physical Function Component Score across disease severity in people with multiple sclerosis**. Error bars represent standard deviations. Higher scores represent more favourable perceived quality of life. Exercisers represent individuals who reported participating in at least two, 30-min exercise sessions·wk^-1^, or had a physical activity score in the leisure domain of the International Physical Activity Questionnaire greater than 600 MET-min·wk^-1^.

**Figure 6:**
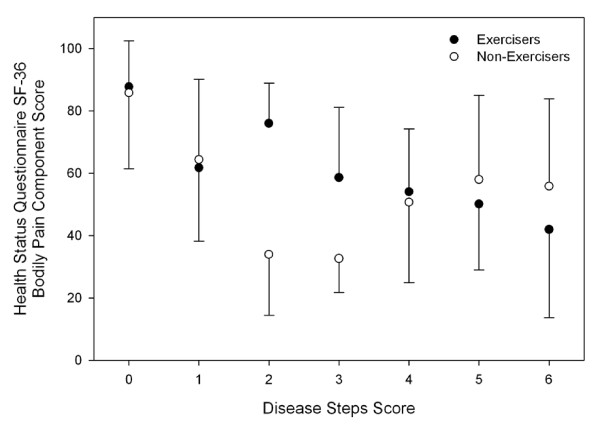
**Health Status Questionnaire Short Form-36 Bodily Pain Component Score across disease severity in people with multiple sclerosis**. Error bars represent standard deviations. Higher scores represent more favourable perceived quality of life. Exercisers represent individuals who reported participating in at least two, 30-min exercise sessions·wk^-1^, or had a physical activity score in the leisure domain of the International Physical Activity Questionnaire greater than 600 MET-min·wk^-1^.

Univariate analysis of variance between disease severity, exercise status and depression, fatigue and QoL scores in persons with MS found main effects for exercise status and disease severity in some QoL and fatigue scores (Table 2). There was an interaction effect for the Physical Function, Bodily Pain and Physical Component Summary Score of the SF36 (Figures 4, 5 and 6).

**Table 2:** Univariate analysis of exercise status, disease severity and quality of life, depression and fatigue scores

	**BDI**	**SF36PF**	**SF36RP**	**SF36BP**	**SFGH**
**Exercise Status**	5.632*	12.983**	1.236	2.149	9.325**
**Disease Severity**	1.159	39.953**	10.437**	4.921**	2.810*
**Interaction effect**	0.822	2.527*	0.988	2.754*	2.004

	**SF36VT**	**SF36SF**	**SF36RE**	**SF36MH**	**SF36PCSS**

**Exercise Status**	5.631*	7.440**	3.074	7.398**	5.532*
**Disease Severity**	3.752**	1.527	0.791	0.839	23.693**
**Interaction effect**	1.191	0.406	0.430	0.450	2.314*

	**SF36MCSS**	**MFISphy**	**MFIScog**	**MFISpsy**	**MFIStot**

**Exercise Status**	5.436*	9.247**	1.153	4.547**	1.549
**Disease Severity**	0.671	10.624**	3.986**	10.489**	7.160**
**Interaction effect**	0.202	0.707	1.813	0.486	0.413

Values represent F score of a univariate analysis of variance. BDI: Becks Depression Inventory, SF36: Health Status Questionnaire Short Form-36, PF: physical function, RP: physical role, BP: bodily pain, GH: general health, VT: vitality, SF: social functioning, RE: emotional role, MH: mental health, PCSS: physical component summary score, MCSS: mental component summary score, MFISphy: Modified Fatigue Impact Scale physical component, MFIScog: Modified Fatigue Impact Scale cognitive component, MFISpsy: Modified Fatigue Impact Scale psychosocial component, MFIStot: Modified Fatigue Impact Scale overall score. IPAQleisure: International Physical

### The influence of the quantity of regular physical activity on fatigue, depression and quality of life scores

Significant but weak correlations between the leisure-time activity subscale of the IPAQ and the Physical Role(r = 0.214) and General Health (r = 0.254) subscales of the SF36, and the Physical (r = -0.220) and Psychosocial (r = -0.246) scales of the MFIS were detected. When overall activity levels were analysed, there were significant correlations between the overall score of the IPAQ and the Physical Function (r = 0.409), Physical Role (r = 0.234), Vitality (r = 0.198) and Physical Component Summary Score (r = 0.312) of the SF36 and the Physical (r = -0.250) and Psychosocial (r = -0.257) scales of the MFIS.

## Discussion

The purpose of this study was to compare fatigue, depression and QoL scores in persons with MS who did and did not participate in regular physical activity. A recent review of exercise intervention studies in persons with MS, found evidence to support the positive effect of exercise on QoL, however these authors concluded their was insufficient research in this area [31]. The findings of this study support the hypothesis that regular physical activity is associated with favourable fatigue, depression and QoL scores in persons with MS.

In the present study, subjects classified as Exercisers reported less fatigue on the Physical and Psychosocial scales and overall score of the MFIS. These results are supported by Trojan et al (2007) who preformed correlation analysis on the General, Mental and Physical scales of the Multidimensional Fatigue Inventory and found that physical activity was weakly correlated with the Physical but not the General or Mental scales of the Multidimensional Fatigue Inventory [32].

Fatigue in MS may be attributed to primary factors related to the disease process or secondary factors such as sleep disturbances, depression, pain and medication use [33]. Theories of primary fatigue in MS include hypo-functioning within the central nervous system [34], reduced glucose metabolism in the cortical regions of the brain [35], reduced inhibition of the primary motor cortex in the pre- and post- exercise period [36] and abnormal cytokine profiles [37,38]. Both aerobic- and resistance-based exercise programs have been found to alter cytokine profiles in MS patients [39,40] and this provides a plausible explanation for the improvement in fatigue seen in some patients following regular physical activity.

Alternatively, improvements in secondary factors such as depression with regular physical activity may explain the improvements seen in fatigue in some MS patients. Depression scores observed in the Exercisers in the present study were significantly lower when compared to Non-exercisers. It is well recognised that exercise is positively associated with psychological well being in the general population [41-45]. It is unclear exactly how exercise improves depression in non-MS populations, however several theories have been proposed including: regulation of the hypothalamic-pituitary axis [46], increased β-endorphin levels [46], normalisation of hippocampal brain derived neurotrophic factor [47], regulation of central monoamines [46] and improved perceptions of self efficacy [48]. The hypothalamic-pituitary axis [49], brain-derived neurotrophic factor [50] and serotonin [51] have all been implicated in MS pathology. If exercise influences hypothalamic-pituitary axis function, brain-derived neurotrophic factor concentration or serotonin concentration in persons with MS, this provides a possible explanation for the decreased incidence of depression observed in persons with MS who regularly participate in physical activity. Alternatively, depression etiology in MS may have a psychological rather than neurobiological explanation. In persons with MS, a positive relationship between activity levels and self-efficacy has been reported [52]. Due to the relatively high incidence of depression in MS, both the etiology and the influence of exercise on depression are areas that warrant further investigation.

In the present study, Exercisers had significantly higher scores on all components of the SF-36, which is suggestive of a higher QoL. These findings are supported by Stuifbergen et al (2006) who found that exercise behaviour, measured using the exercise/physical subscale of the Health Promoting Lifestyle Profile II, was positively associated with QoL[7]. When QoL was assessed across the spectrum of disease severity, we found interaction effects between exercise status and disease severity for the Physical Function, Bodily Pain and Physical Component Summary Scores of the SF36 (Figures 4, 5 and 6). Visual interpretation of these figures suggests that although these scales were similar between Exercisers and Non-exercisers with mild MS (DSS ≤1), regular physical activity impacts favourably upon QoL in patients with a DSS between two and four. Once disease severity reached a DSS ≥5, QoL was again similar between Exercisers and Non-exercisers. Therefore, participation in regular physical activity appears to have the greatest positive influence on QoL in patients once visual abnormalities in gait have developed, until the point of time when patients become cane-dependant.

To date the majority of exercise intervention studies have focused on patients with mild-moderate MS, and although these studies have been associated with positive benefits for persons with MS [53,54], little information is available on the influence of physical activity for persons across the disease spectrum. The results of this study suggest the exercise may have a greater effect on QoL in the physical domain in persons with moderate MS. The reason for this is unknown, however it may be speculated that regular physical activity improves a patients' ability to perform physical tasks, or improves a patients' perception of the impact their disability has on their physical functioning. This improved QoL in the physical function, and physical component summary scores of the SF36, may be particularly evident in patients with moderate MS. Perhaps in persons with mild MS, the physical limitations of the disease are minimal and therefore irrespective of exercise status the impact of the disease on QoL in the physical domain is minimal. Similarly, it maybe once disease severity and physical limitation become severe, that these limitations will significantly impact on QoL irrespective of exercise status. This is an area that warrants further investigation, in order for health care professionals to implement exercise intervention and management programs to those patients who will receive maximal benefit.

Cross-sectional studies investigating the role of physical activity on QoL in persons with MS have typically correlated activity levels and QoL scores [7,55]. The present study reported significant weak correlations between both leisure and overall activity scores on the IPAQ, and some fatigue, depression and QoL scores in persons with MS. This suggests that there may not be a linear relationship between activity levels and fatigue, depression and QoL scores in persons with MS.

The current study classified Exercisers as subjects who completed at least two 30-min exercise sessions·wk^-1^. This exercise volume does not meet the recommended dose of exercise prescribed by the American College of Sports Medicine [56]. It is unknown whether the completion of two 30-min exercise sessions·wk^-1 ^would elicit adaptations in cardiovascular fitness or reduce the risk of co-morbidities and unhealthy weight gain in this clinical population. However, the current study does suggest that completing at least two 30-min exercise sessions·wk^-1 ^may positively influence disease specific symptoms such as fatigue and depression in persons with MS.

Due to the cross-sectional nature of this study we cannot provide definitive conclusions on the direction of causality between activity levels and fatigue, depression and QoL scores. Subjects in this study were recruited from two separate patient databases. Although we have no reason to believe there would be any differences in the disease characteristics of the patients in the two databases utilised in this study we did not perform any analysis to confirm this. Data was collected in a de-identified manner and we were unable to provide information on responders vs. non-responders and we cannot guarantee that the sample population utilised in this study provides an accurate representation of all persons with MS living in Queensland, Australia. This study had a reasonably low response rate (31%) and it is possible that health conscious individuals may have been more inclined to participate in the study. This may be demonstrated by the relatively higher number of Exercisers in this study (43%) compared to a rate of 28.6% in a recent cross-sectional survey of men with MS [57]. Additionally it is worthwhile noting that although the sex distribution between subjects classified as Exercisers (23% male) and Non-exercisers (17% male) are not statistically significant, these numbers may in fact represent a difference that may impact on the results of this study.

Subjects recruited through the MS Society of Queensland's database had not had their MS diagnosis confirmed by a physician or neurologist, and disease course and severity are patient and not physician reported, this may provide a source of error in the patient characteristics reported. The final limitation to consider when interpreting the results of this study is that we found a statistically significant difference in the MSIS-29 score between Exercisers and Non-exercisers. The MSIS-29 measures the impact of the disease on the individual of the previous 2 weeks, it is possible that regular exercise may improve the MSIS-29 by improving either the perception or the ability of the individual to perform physical tasks, alternately, this difference in MSIS-29 score may indicate a difference in the baseline characteristics between the Exercisers and Non-exercisers in this study.

The strength of the current study is that although exercise intervention studies have been associated with improved fatigue, depression and QoL in small samples of MS patients, this study provides an overall view of these relationships. This study focused on patients across the entire disease spectrum and was not limited to those with mild-moderate MS.

## Conclusion

In summary, subjects who participated in regular physical activity reported better results on the BDI, all scales of the SF36, and some scales of the MFIS. This suggests that persons with MS who regularly participate in physical activity have favourable fatigue, depression and QoL scores, when compared to persons with MS who do not regularly participate in physical activity. This study gives strength to previous suggestions that regular physical activity may improve fatigue, depression and QoL in persons with MS.

This study reinforces that health care providers should promote physical activity in persons with MS as a strategy to improve QoL. This study does highlight the need for exercise intervention studies to occur not only in persons with mild-moderate disability but in those patients with moderate-severe disability as well, in order to understand the potential for physical activity to improve QoL in all persons with MS. Further research investigating the mode of exercise that will provide maximum benefit to persons with MS, across the entire disease spectrum is warranted.

## Abbreviations

*MS*: multiple sclerosis; *Exercisers*: persons with MS who regularly participate in at least two, 30 min exercise sessions per week; *Non-exercisers*: persons with MS who do not regularly participate in at least two, 30 min exercise sessions per week; *QoL*: quality of life; *DSS*: Disease Steps Scale; *MSIS-29*: Multiple Sclerosis Impact Scale; *IPAQ*: International Physical Activity Questionnaire; *MET*: metabolic equivalent; *SF36*: Heath Status Questionnaire Short Form 36; *BDI*: Beck's Depression Inventory; *MFIS*: Modified Fatigue Impact Scale

## Competing interests

The authors declare that they have no competing interests.

## Authors' contributions

NS was involved in all aspects of this study. She was involved in the concept and design, data collection and collation, data analysis, writing and editing of the manuscript. CM was involved in the conception and design of the study, as well as data analysis and writing and editing of the manuscript. Both authors read and approved the final manuscript.
